# Structural Characterization of a Water-Soluble Polysaccharide from the Fruiting Bodies of *Agaricus bisporus*

**DOI:** 10.3390/ijms15010787

**Published:** 2014-01-08

**Authors:** Jinzhe He, Anqiang Zhang, Qiaomei Ru, Dandan Dong, Peilong Sun

**Affiliations:** 1 College of Biological and Environmental Engineering, Zhejiang University of Technology, Hangzhou 310032, China; E-Mails: hejzgd@163.com (J.H.); zhanganqiang@zjut.edu.cn (A.Z.); 2 Yuhang District Agricultural Monitoring Centre of Hangzhou, Hangzhou 311199, China; E-Mails: ruqiaomei@zjut.edu.cn (Q.R.); ranranbudong@aliyun.com (D.D.)

**Keywords:** *Agaricus bisporus*, polysaccharide, structural characterization, NMR spectrum

## Abstract

An edible fungal polysaccharide termed as *ABP* was obtained by extraction with hot water, and followed successive chromatographic purification using DEAE-Sepharose Fast Flow column and Sephacryl S-300 High-Resolution column. A symmetrical peak was obtained on high-performance size-exclusion chromatography with an average molecular weight of 5.17 × 10^4^ Da, which was named *ABP*, and its main components were d-glucose and d-mannose. Based on the study of methylation analysis, along with FT-IR, GC, GC-MS, 1D ^1^H and ^13^C NMR and 2D NMR (H-HCOSY, TOCSY, HMQC, and NOESY), its chemical structure was featured with a repeating unit (1→6) linking β-d-Glc*p* as the main backbone with (1→4)-linked α-d-Man*p* units. The structure of the mainly repeating units of *ABP* was established as:

→6)-β-D-Glucp-(1→4)-α-D-Manp(1→6)-β-D-Glucp-(1→6)-β-D-Glucp-(1→

## 1. Introduction

For thousands of years, mushrooms have been known as a source of medicine [1], many studies showed that polysaccharides were the main occurring biological constituents in common mushrooms. Polysaccharides are highly appreciated for their multiple biological and pharmacological activities such as antitumor, immune-modulating, anti-inflammatory, anti-atherogenic and hypoglycemic actions [2,3]. The polysaccharides from several species, such as *Ganoderma*, *Lentinus*, *Agaricus* or *Flammulina*, have been widely studied [4–6]. Some structural patterns of polysaccharides have been found from various mushrooms. Many evidences have indicated that the biological activities of polysaccharides depended on their structural features such as the compositions of sugar, type of glycosyl linkage, branch structures, molecular weight and concentration [7–9]. In this field, the β-(1→3), (1→6) glycosidic linkages are supposed to play an important role in enhancing the antitumor and the immunomodulatory effects [10–14]. Furthermore, other structural features, such as the (1→4), (1→6) moiety found in several fungal species, have also been demonstrated to enhance the immune system [15].

*Agaricus bisporus* (*A. bisporus*) is one of the most favorable mushrooms in the world market, and has been well characterized by not only its delicious taste and high protein content, but also the high content of dietary fiber and functional compounds, and is also considered to have immense potential as a source of valuable medicinal compounds. We have previously reported the antioxidant property, monosaccharide composition and molecular weight distribution of a water soluble crude polysaccharide from *A. bisporus* [16]. As part of a continuing investigation of the chemical structures and biological activities of the polysaccharides from this fungus, in this manuscript, we report the isolation and structural characterization of *A. bisporus* polysaccharide (*ABP*) from *A. bisporus*.

## 2. Results and Discussion

### 2.1. Isolation and Purification

**Figure 1.**
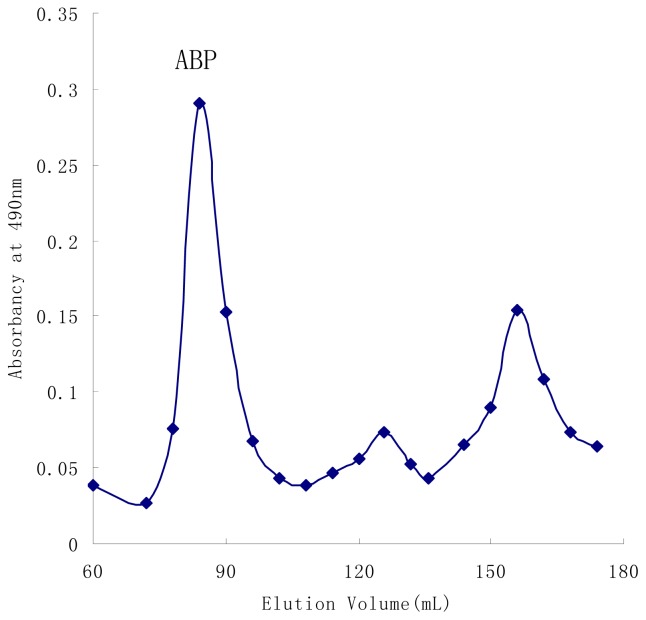
The elution of *ABP* isolated from the fruiting bodies of *A. bisporus* by gel-permeation chromatography on Sephacryl S-300 High-Resolution column.

**Figure 2.**
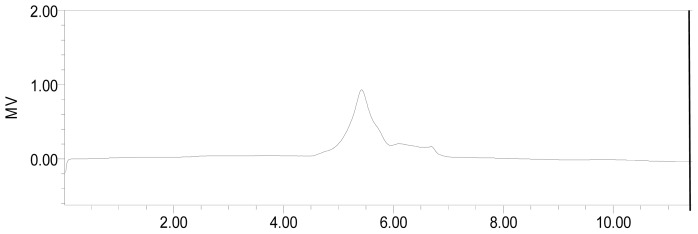
The HPSEC of purified polysaccharide *ABP* (retention time).

The water-soluble polysaccharide from the fruiting bodies of *A. bisporus* was purified by DEAE-Sepharose Fast Flow column and gel-permeation chromatography on Sephacryl S-300 High-Resolution column (XK 2.6 × 100 cm). Figure 1 showed the elution pattern of *ABP*. High-performance size-exclusion chromatography (HPSEC) gave a single symmetrical peak (Figure 2) with exclusion from the TSK gel G4000PWXL column, indicating that *ABP* was a homogeneous polysaccharide. The UV scanning found no absorption at both 260 and 280 nm and thus suggested the polysaccharide contained no nucleic acids and protein. The total sugar content of *ABP* was estimated as 99.1% by phenol-sulfuric method [17], and sugar compositional analysis indicated that sugar residues were composed of d-glucose, d-mannose, d-galactose and d-xylose in the molar ratio of 2.25:2.00: 0.35:0.20.

The weight-average molar mass of *ABP* was estimated to be 5.17 × 10^4^ Da by the following equation: lg *M*_w_ = 11.5 − 0.376 *V*_t_ (*R*^2^ = 0.972), which was from the calibration curve of molecular size distribution with automatically calculated by GPC software from HPLC, and where *M*_w_ was the molecular weight of polysaccharide and *V* was the retention volume of polysaccharide.

### 2.2. Structural Characterization of *ABP*

The inter-glycosidic linkages between monosaccharide residues of *ABP* were investigated by methylation analysis. The polysaccharide was methylated three times, followed by hydrolysis and alditol acetate preparation. The complete methylation was confirmed by the disappearance of the hydroxyl peak (3200–3700 cm^−1^) in IR spectrum. According to sugar analysis of acetylated methyl glycosides and the GC-MS analysis of the alditol acetates, hydrolysates of *ABP* showed following methylated sugar derivatives: (I) 2,3,4-tri-*O*-methylglucose; (II) 2,3,6-tri-*O*-methylmannose; (III) 2,3,4-tri-*O*-methylgalactose and (IV) 2,3,4-tri-*O*-methylxylose in a molar ratio of 2.25:2.00:0.35:0.20, respectively, as summarized in Table 1.

These results indicated that the backbone chains of (1→6) linked-d-glucopyranosyl unit and (1→4) linking non-reducing-end mannopyranosyl unit were mainly present in the polysaccharide, and the component (III) and (IV) contained a relatively low amount of (1→6) linking mannopyranosyl and galactopyrannosyl residues. However, methylation analysis was not sufficient to distinguish different surroundings of residues with α or β linkage pattern. Some linkage patterns of sugar residues and structural characterizations need to be further confirmed by ^1^H, ^13^C and 2D NMR experiments.

**Figure 3.**
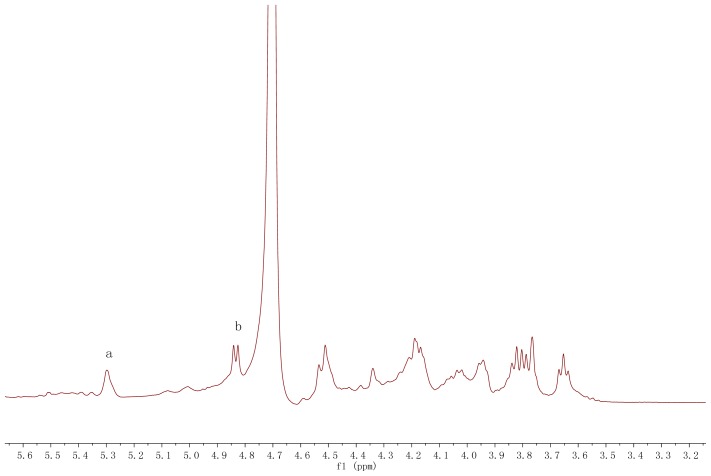
500 MHz ^1^H NMR spectrum of the *ABP* isolated from fruiting bodies of *A. bisporus*.

A series of ^1^H and ^13^C and 2D-(H-HCOSY, TOCSY, HMQC, NOESY) NMR experiments were employed to assign the proton and carbon signals of the main residues of *ABP*. The ^1^H NMR spectrum (Figure 3) of the polysaccharide clearly showed two resonances of equal intensity in anomeric regions at δ 5.29 and δ 4.83, respectively. The sugar residues were correspondingly designated as a and b with other sugar protons in the regions of δ 3.50–δ 4.52. These characteristic signals were corresponding to the main parts of two sugar residue structures from mannose and glucose (residues a and b). The NMR signals of galactose and xylose were no indication owing to their low relative area and poor nuclear magnetic resonance characterizations.

**Figure 4.**
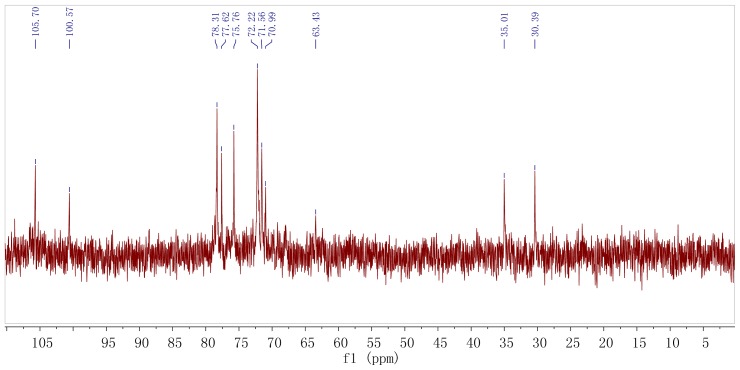
^13^C NMR spectrum of the *ABP* isolated from fruiting bodies of *A. bisporus*.

**Figure 5.**
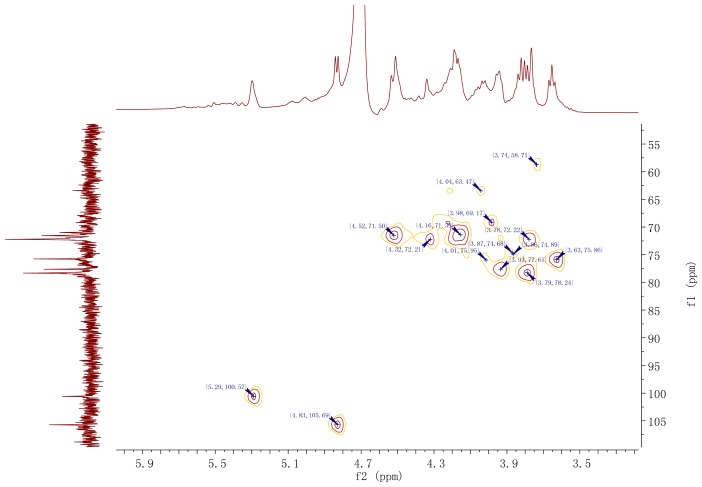
HMQC NMR spectrum of the *ABP* isolated from fruiting bodies of *A. bisporus*.

The ^13^C NMR spectrum (Figure 4) of the polysaccharide showed the presence of two main anomeric carbons at δ 100.6 and δ 105.7 (corresponding to residues a and b), respectively, with other sugar ring carbons linked to oxygen atoms in the region of δ 63.4–δ 78.3. Characteristic fatty acids signals were identified as CH_2_–CH_2_–COOR (35.01 ppm), and a CH_2_-group at 30.39 ppm. The HMQC spectrum (Figure 5) showed two dominant cross-peaks at δ 5.29/100.6 and δ 4.83/105.7 corresponding to hexopyranosyl residues in the region of anomeric resonances, indicating that there were two main sugar residues in the polysaccharide. The ^13^C NMR was in agreement with the above ^1^H NMR analysis.

Residue a had an anomeric signal at δ 5.29. Cross-peaks at δ 5.29/4.51 and δ 4.51/4.16 were detected in ^1^H–^1^H COSY spectrum, and since δ 5.29 corresponded to H-1, the δ 4.51 and δ 4.16 signals were assigned to H-2 and H-3, respectively. The ^1^H resonances for H-4 and H-5 in residue a were assigned from the cross-peaks in ^1^H–^1^H COSY, TOCSY, NOSEY and HMBC spectra. The H-6a and H-6b resonances were obtained from ^1^H–^1^H COSY and TOCSY spectra. The carbon signals from C-1 to C-6 of residue a were identified from the HMQC spectra (Figure 4). The manno-configuration for residue a was supported from relatively small coupling constant value of *J*_H-1, H-2_ < 3 Hz and large coupling constant values of *J*_H-3, H-4_ (7.5 Hz), *J*_H-4, H-5_ (10 Hz) and *J*_C-1, H-1_ value (166 Hz) [18–20]. Residue a showed the down-field anomeric H-1 signals (H > δ 5.0) and the anomeric C-1 signal at δ 100.6, but did not provide information about glycosidic type linkage. The anomeric chemical shifts of mannose did not allow unambiguous determination of mannose linking configuration [21]. A NOESY experiment revealed inter-residue correlations between H-1 and H-2, indicating that residue a was α-configuration [22]. Additionally, the chemical shifts of the corresponding carbons were revealed from the HMQC spectrum with ^13^C resonances assigned in Table 2. The carbon shifts of the residues a showed C-1 at δ 100.6; C-2, δ 71.4; C-3, δ 75.9; C-4, δ 77.4; C-5, δ 74.7; C-6, δ 63.4. The downfield carbon chemical shift C-4 at δ 77.4 was reasonably assigned to the substituted C-4 as a result of the *O*-replace effect, which indicated the substitution of residue a at the C-4 position and the existence of (1→4)-linkage. The signal at δ 63.4 was assigned to the unsubstituted C-6, indicating that residue a was →4)-linked-α-d-Man*p*-(1→. The result was inconsistent with the GC-MS data for this linkage.

The proton chemical shifts of H-1 to H-6 from residue b were readily assigned by the ^1^H–^1^H COSY spectrum and further confirmed by the TOCSY spectrum. Magnetization relayed well through the spin system, as expected for the glucose-configuration, and all cross-peaks were clearly visible. The high-field H-1 signals (H < δ 5.0), the deshielded anomeric signal C-1 at δ 105.7 and the distinct splitting peak on the residue b revealed that the units of sugar residue b had β-configuration linkage, which was confirmed by *J*_H-1, H-2_ values (7.5 Hz) [23]. Large coupling constants of *J*_H-2, H-3_ and *J*_H-3, H-4_ (9–10 Hz) indicated that residue b was a glucose-configuration [24,25], and presented as β-d-glucose glycosidic linkage. The carbon signals from C-1 to C-6 for residue b were identified from the HMQC spectrum. The glycosidic linkages of the residue b showed C-1 at δ 105.7; C-2, δ 73.6; C-3, δ 75.3; C-4, δ 72.0; C-5, δ 78.2; C-6, δ 68.9, respectively (Table 2). As a result of the glycosylation effect, the signal for C-6 of residue b was shifted downfield to δ 68.9, which indicated that residue b was substituted at C-6 position. The result suggested the presence of a (1→6) linkage glucose residue in the polysaccharide, and was identified as (1→6)-substituted β-d-glucosepyranoside. The above results were in agreement with data obtained from the sugar and methylation analysis of *ABP*.

The glycosidic linkages between the two residues were determined by the observed inter-residue connectivities generated by NOESY and HMBC experiments. Inter-residue NOEs connectivities were observed between H-1 of residue a and H-6 of residue b, between H-1 of residue b and H-6 of residue b, HMBC spectrum showed clear correlations between H-1 of residue a and C-6 of residue b, between H-1 of residue b and C-4 of residue a, between H-1 of residue b and C-6 of residue b. Based on the data presented above, it demonstrated that the mainly repeating units for *ABP* with the following structure:

→6)-β-D-Glucp-(1→4)-α-D-Manp(1→6)-β-D-Glucp-(1→6)-β-D-Glucp-(1→

## 3. Experimental Section

### 3.1. Materials and Methods

Fruiting bodies of *A. bisporus* (*Yingxiu 1^#^*) were collected from Zhejiang Agriculture Research Institute in Zhejiang province, China. T-series dextrans were purchased from Wuhan Putus Macromolecular Sci. & Tech. Co. Ltd., (Wuhan, China). Monosaccharide standards (d-galactose, d-arabinose, l-fucose, l-rhamnose, d-manonose, d-xylose, d-glucose and erythrose), trifluoroacetic acid (TFA) and dimethyl sulfoxide (DMSO) were purchased from Sigma (St. Louis, MO, USA). All other chemical reagents were of grade AR from Shanghai Chemical Co. (Shanghai, China). DEAE-Sepharose Fast Flow and Sephacryl S-300 High Resolution were purchased from Amersham Pharmacia Biotech (Uppsala, Sweden). TSKgel G4000PWXL column was purchased from Shanghai Biological Technology Co., Ltd. (Shanghai, China). Ultrahydrogel TM 120 and 1000 (78 × 300 mm) columns were purchased from Waters Co., Milford, MA, USA. HPLC was carried out on a waters 1525 HPLC system (1525 HPLC pump, 2414 refractive index detector; Waters Co., Milford, MA, USA).

### 3.2. Extraction of Crude Polysaccharides

The fruiting bodies of *A. bisporus* were dried at 50 °C for 48 h, and ground to obtain fine powder (40 meshes). The powder of fruiting bodies was extracted with 25 times’ volume of boiling water for 2 h. After filtration, the residues were re-extracted twice in the same way. The liquid extracts were combined and concentrated into one-fifth of the original volume under vacuum, and 95% ethanol was added slowly to a final concentration of 80% and kept at room temperature overnight. The precipitate was obtained by centrifugation (6000 rpm, 15 min, 4 °C), then washed three times with anhydrous ethanol, acetone and ether, and finally was lyophilized to obtain water soluble crude polysaccharides of *A. bisporus*.

### 3.3. Purification of Crude Polysaccharides

The crude polysaccharide was redissolved in deionized water and using a DEAE-Sepharose Fast Flow column (XK 2.6 × 100 cm), eluted first with water, followed stepwise by 0–1.0 M NaCl. The fractions were collected by a fraction collector and compounds were detected by the phenol-sulfuric method, then the fraction corresponding to major sugar peak was further purified, using gel-permeation chromatography on Sephacryl S-300 High-Resolution column (XK 2.6 × 100 cm) with distilled water as eluent at 0.6 mL/min. The sample was collected by 6 mL/tube using an auto-collector, and the main sugar peaks were collected and lyophilised to get a white homogeneous *A. bisporus* polysaccharide (*ABP*).

### 3.4. Methylation Analysis and Monosaccharide Composition Analysis

The polysaccharide *ABP* was treated three times by NaOH-DMSO-MeI method [26]. The reaction mixture was extracted with CHCl_3_, and the solvent was then removed by evaporation. Complete methylation was confirmed by the disappearance of the OH band (3200–3700 cm^−1^) in IR spectrum. The methylated polysaccharide sample (4.0 mg) was hydrolyzed in a long tube with 6 mL of 2 M trifluoroacetic acid (TFA) at 110 °C for 2 h, and the excess acid was removed by azeotropic distillation with methanol. The resulting hydrolysates were reduced by NaBH_4_ (25 mg) and acetylated with acetic anhydride, then distilled with methanol to remove excess boric acid, followed by drying over P_2_O_5_. Monosaccharide compositions were analyzed by an Agilent 7890 A gas chromatograph system was equipped with a flame ionization detector (FID) using a DB-1701 capillary column (30 m × 0.25 mm × 0.25 μm). The methylated alditol acetates were analyzed by chromatography-mass spectrometry (GC-MS) using a Finnigan Trace Ultra-DSQ II (Thermo Co., Austin, TX, USA) system equipped with a TG-5MS column (30 m × 0.25 mm × 0.5 mm; Thermo Co., Austin, TX, USA). The column temperature was initially set at 120 °C, then programmed from 120 to 240 °C at a rate of 5 °C/min and held at 240 °C for 30 min. The split ratio was 1:25 and the injection volume was 1.0 μL. The injector and the detector temperatures were both set at 250 °C.

### 3.5. Determination of Purity and Molecular Weight

Determinations of the homogeneity and the molecular weight of the samples were done by high-performance size-exclusion chromatography (HPSEC), using a Waters 1525 HPLC system fitted with TSKgel G4000PWXL column (Sigma-Aldrich Co., Japan) and two serially linking Ultrahydrogel TM 120 and 1000 (78 × 300 mm) columns (Waters Co., Milford, MA, USA), respectively, a Waters 2414 RI detector (Waters Co., Milford, MA, USA), eluting with 0.01 M NaNO_3_ at pH 7.0 with a flow rate of 0.8 mL/min. The column was kept at 30.0 ± 0.1 °C. The linear regression was calibrated by T-series dextrans known molecular mass (T-110, 80, 70, 40, 25, 10) as standard. A calibration curve was prepared by plotting *V*_e_/*V*_0_ (elution volume/void volume, mL) *versus* log molecular weight and molecular weight of the unknown polysaccharide was determined.

### 3.6. Spectroscopic Methods

Fourier-transform infrared (FT-IR) spectra was recorded from 4000 to 400 cm^−1^ with a 6700 Nicolet Fourier transform-infrared spectrophotometer (Thermo Co., Madison, WI, USA), using films prepared by the dried polysaccharides and KBr pellets.

### 3.7. Nuclear Magnetic Resonance (NMR) Analysis

The purified polysaccharide sample (20 mg) was deuterium-exchanged three times by lyophilization with D_2_O and then dissolved in D_2_O (99.9%, 0.5 mL). ^1^H NMR (25, 60 °C) and ^13^C NMR (25 °C) spectra were determined in 5-mm tubes using a Broker ANANC III (500M) spectrometer (Bruker Co., Fallanden, Switzerland). ^1^H chemical shifts were referenced to the HDO resonance at δ 4.78 (25 °C) as internal standard. ^13^C chemical shifts were determined in relation to DSS (sodium 2,2-dimethyl-2-5-sulfonate, δ 0.00) as external calibration. ^1^H–^1^H correlated spectroscopy (COSY), total correlation spectroscopy (TOCSY) and heteronuclear multiple quantum coherence (HMQC) were used to assign signals. Two-dimensional heteronuclear multiple-bond correlation spectroscopy (HMBC) and two-dimensional nuclear overhauser enhancement spectroscopy (NOESY) were used to assign inter-residue linkages and sequences.

## 4. Conclusions

In this study, we obtained a homogeneous polysaccharide from the fruiting bodies of *A. bisporus* by DEAE-Sepharose Fast Flow column (XK 2.6 × 100 cm) and gel-permeation chromatography on Sephacryl S-300 High-Resolution column (XK 2.6 × 100 cm), and identified its structural characteristics by UV, FT-IR, GC-MS, methylation analysis, ^1^H and ^13^C NMR spectrum (including COSY, TOCSY, HMQC, HMBC and NOESY). The results indicated that *ABP* was mainly composed of glucose and mannose with the molecular weight 5.17 × 10^4^ Da, and its structure was featured with a repeating unit (1→6) linking β- d-Glc*p* as main backbone with (1→4)-linked α- d-Man*p* units. In most fungi examined, polysaccharides consisting of β-(1→3), β-(1→6)-d-Glc*p*, and α-(1→3)-d-Man*p* have been found in many mushroom-derived polysaccharides cultivar, and reported to be the major components of the cell wall and the intercellular matrix [27–31]. However, the structural characterization containing (1→6) linking β-d-Glc*p* as main backbone with (1→4)-linked α-d-Man*p* units of *ABP* has not been previously reported, and *ABP* is therefore a novel fungal polysaccharide.

## Figures and Tables

**Table 1.** GC-MS data for methylation analysis of *ABP* isolated from the fruiting bodies of *A. bisporus*.

Methylated sugar	Linkage type	Molar ratio	Major mass fragment (*m*/*z*)
2,3,4-tri-*O*-methylglucose	→6)-d-Glc*p*-(1→	2.25	43, 87, 101, 117, 129, 161, 189, 233
2,3,6-tri-*O*-methylmannose	→4)-d-Man*p*-(1→	2.00	43, 87, 101, 117, 129, 161, 189, 261
2,3,4-tri-*O*-methylgalactose	→6)-d-Gal*p*-(1→	0.35	43, 87, 101, 117, 129, 161, 173, 189, 233
2,3,4-tri-*O*-methylxylose	→6)-d-Xyl*p*-(1→	0.20	87, 99, 117, 139, 161, 217, 233

**Table 2.** Chemical shift data for *ABP* isolated from the fruiting bodies of *A. bisporus*.

Residue	Proton or carbon (^1^H/^13^C)						

		1	2	3	4	5	6a 6b
→4)-α-d-Man*p* (1→ (a)	H	5.29	4.51	4.16	3.93	3.87	4.04 3.89
	C	100.6	71.4	75.9	77.4	74.7	63.4
→6)-β-d-Glc*p* (1→ (b)	H	4.83	3.63	4.02	3.78	3.79	3.98
	C	105.7	73.6	75.3	72.0	78.2	68.9

The NMR signals of galactose and xylose were no indication owing to their poor nuclear magnetic resonance characterizations.
